# Wheat Albumin Increases the Ratio of Fat to Carbohydrate Oxidation during the Night in Healthy Participants: A Randomized Controlled Trial

**DOI:** 10.3390/nu11010197

**Published:** 2019-01-18

**Authors:** Shinichiro Saito, Toshitaka Sakuda, Aiko Shudo, Yoko Sugiura, Noriko Osaki

**Affiliations:** 1 Biological Research Laboratories, Kao Corporation, 2-1-3 Bunka Sumida-ku, Tokyo 131-8501, Japan; sakuda.toshitaka@kao.com (T.S.); osaki.noriko@kao.com (N.O.); 2 Health Care Food Research Laboratories, Kao Corporation, 2-1-3 Bunka Sumida-ku, Tokyo 131-8501, Japan; shudou.aiko@kao.com (A.S.); sugiura.yoko@kao.com (Y.S.)

**Keywords:** energy expenditure, fat oxidation, human, respiratory quotient, wheat albumin

## Abstract

Not only are energy expenditure (EE) and the respiratory quotient (RQ) parameters of the energy nutrient utilization and energy balance, they are also related to the development of obesity. In this study, post-meal night-time energy metabolism was investigated following the oral ingestion of wheat albumin (WA) with a late evening meal. A randomly assigned, double-blind, placebo-controlled crossover trial for a single oral ingestion in healthy participants was completed. The participants ingested the placebo (PL) or WA (1.5 g) containing tablets 3 minutes before the late evening meal at 22:00 hour, and energy metabolism was measured using a whole-room indirect calorie meter until wake-up. The participants were in bed from 00:00 hour until 06:30 hour. Twenty healthy participants completed the trial and were included in the analyses. Night-time RQ and carbohydrate oxidation were significantly lower following the WA treatment as compared with the PL treatment. Although the total EE was not significantly different between treatments, postprandial fat oxidation was significantly higher following the WA treatment as compared with the PL treatment. In conclusion, WA has the potential to shift the energy balance to a higher ratio of fat to carbohydrate oxidation during the night.

## 1. Introduction

Obesity is well known to result from an imbalance between energy consumption and expenditure. Therefore, increases in physical activity or proper diet therapy are recommended to maintain body weight. In addition, recent studies have reported that a lower respiratory quotient (RQ) or ratio of fat to carbohydrate oxidation was associated with weight gain over several years in non-diabetic Pima Indians [1] and non-obese Italian women [2], even after the adjustment for energy expenditure (EE), suggesting that the RQ is an independent predictor against the development of obesity. Moreover, in individuals with obesity, carbohydrate oxidation did not change while fat oxidation was attenuated by aging [3]. Thus, in addition to the imbalance between energy consumption, a lower ratio of fat to carbohydrate oxidation is a risk factor for weight gain.

A previous study showed that wheat albumin (WA), which has a long history of human consumption as a natural food constituent, is a potential agent against postprandial hyperglycemia [4]. As carbohydrate overloading stimulates carbohydrate oxidation together with fat storage and reduced fat oxidation [5], we hypothesized that carbohydrate loading during the night would induce a lower ratio of fat and carbohydrate oxidation, even in healthy individuals, presumably due to the lower physical activity during the night. Based on our unpublished pilot study, we predicted that this phenomenon would be improved by WA via its suppressive effect on the glucose response after meals.

Thus, we investigated energy metabolism during the night with or without a single oral ingestion of WA with a late evening meal.

## 2. Materials and Methods 

### 2.1. Ethics Approval and Consent to Participate

This study was performed in accordance with the tenets of the Declaration of Helsinki (2013) and was approved by the Ethical Committee of the Kao Corporation (Tokyo, Japan). After receiving a full explanation of the study, all participants provided written informed consent. The study was registered with the University Hospital Medical Information Network (UMIN) clinical registry prior to the enrolment of the first participant as UMIN000020151; registered 18 December 2015 at http://www.umin.ac.jp/ctr/index.htm.

### 2.2. Study Design

**Figure 1:**
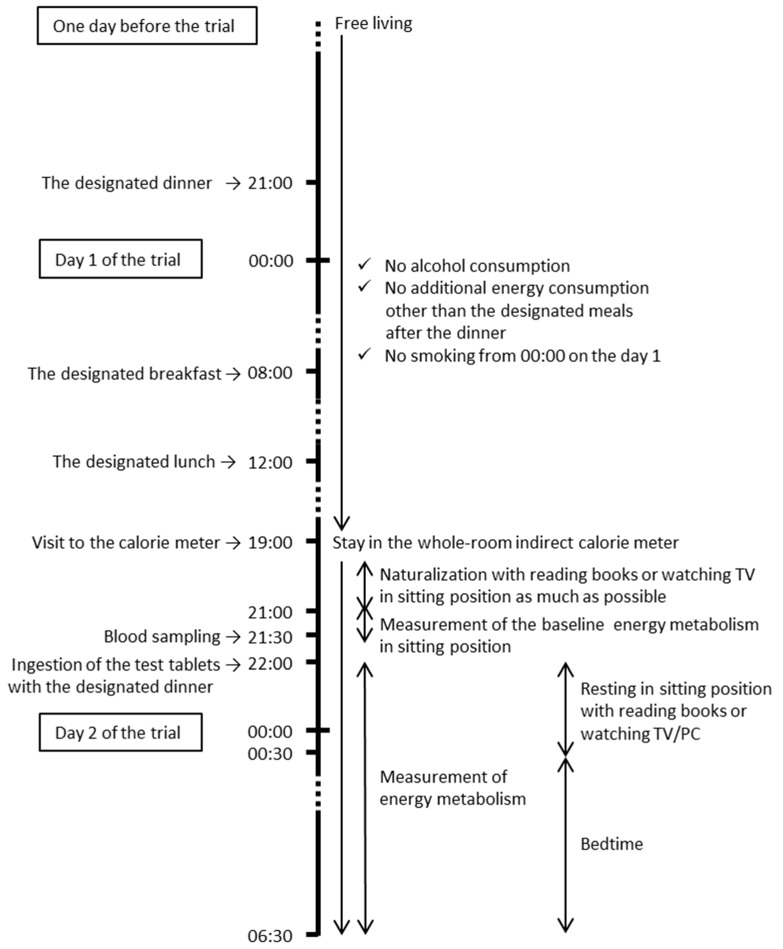
The study protocol.

This study was a randomized, double-blind, placebo-controlled, crossover trial performed under supervision by the physician in charge with a 5-day washout period. The study protocol is shown in Figure 1. Between the screening and the second visit, the participants were instructed to maintain and record their dietary, alcohol, and smoking habits, and level of physical activity. The participants were prohibited from drinking alcohol and undergoing heavy exercise the day before the visits and from smoking cigarettes on the days of the visits based on the report showing that the energy expenditure was increased by exercise or smoking [6]. The participants consumed a designated meal (3310 kJ, protein (P):fat (F):carbohydrate (C) = 14:25:61 as the energy value) for dinner in the evening until 21:00 hour one day before the trial, and had a designated breakfast (2249 kJ, P:F:C = 11:51:38 as the energy value) at 08:00 hour and designated lunch (2887 kJ, P:F:C = 17:18:65 as the energy value) at 12:00 hour on the day of the trial. The participants were not allowed any energy intake other than the designated meals from after dinner on the day before the visit until after completion of the visit. After bathing, the anthropometric parameters of the participants, such as body weight and blood pressure, were measured, as shown in Table 1, and then they entered the whole-room indirect calorie meter (chamber room) at 19:00 hour and underwent low-energy activities such as watching TV, using the PC, or reading books in a sitting position until 21:00 hour to habituate to the conditions of the chamber room (25 °C, 40% humidity). At 21:00 hour, the participants’ baseline energy metabolism levels were measured in a sitting position for 30 minutes. Blood samples were obtained at 21:30 hour via the specific window on the chamber door to collect samples with no air exchange between the inside and outside of the room. The participants ingested PL or WA (1.5 g) containing tablets 3 minutes before the designated late evening meal (Japanese style meal, such as rice, grilled fish, grilled chicken, and boiled vegetables; 3423 kJ, P:F:C = 16:19:65 as the energy value) at 22:00 hour and their energy metabolism was measured until wake-up. The participants slept from 00:30 hour until 06:30 hour and their sleep quality was measured using an Actigraph (ActiGraph, Pensacola, FL, USA). Water consumption was controlled during the measurement period. The treatment allocation was concealed throughout the study (from screening to finalizing the dataset) from the people involved, including the participants, the caregivers, the physicians, the manufacturers of the test tablets, the person in charge of the allocation, and the outcome assessors.

### 2.3. Participants

A sufficient sample size for the primary outcome of postprandial fat oxidation was estimated to be 20 participants based on the outcomes of our unpublished pilot study outcome (power 0.8 and type I error 0.05). In this study, potential participants were screened from men and women aged from 24 to 59 years. Participants were excluded if they met the following exclusion criteria: (1) Presence of liver, kidney, or heart disease; respiratory, endocrine, metabolism, nervous system, or consciousness dysfunction; diabetes; or other diseases; (2) previous experience of seizures based on circulatory-diseases or under treatment for a condition of this type; (3) presence of arrhythmia; (4) taking medications for hyperglycemia, lipidemia, or hypertension; (5) experience of seizures based on a neural disease; (6) surgery within two months before the trial; (7) previous gastrectomy or enterectomy; (8) allergies to any constituents in the test meal or tablets; (9) unpleasant feeling during blood drawing; (10) claustrophobia; (11) insomnia; (12) chronic headache; (13) donation of 200 mL or more of blood within one month before the trial or 400 mL or more of blood within three months before the trial; (14) a habitual bed-time of after 1:00 a.m. on weekdays; (15) heavy smoker (>20 cigarettes); (16) weight change of more than 2.0 kg one month prior to informed consent; (17) shift worker or engaged in night work and shift operations three months prior to informed consent; (18) pregnant or expecting pregnancy; (19) taking supplements or food for a specific use of health authorized by the government; (20) were not checked by a doctor regarding their health condition or their health examination results for the past two years prior to the trial could not be accessed; (21) did not disclose their age and their latest health examination result; (22) did not reply to the questionnaire about living situation and condition; and (23) could not indicate their menstruation situation. The participants were randomly assigned to each sequence (ingestion order) with stratified randomization for glucose, triglyceride, age, and sex using computer-generated random numbers under blind conditions. 

### 2.4. Test Tablets

The test diet was three tablets containing a total of 1.5 g WA for a single oral administration. The PL tablets did not contain any WA. These were prepared using identical ingredients including flavors and preservatives except for the WA, with a weight of 1.1 g for each tablet. The energy value of a single dose was 14.7 kJ for the WA tablet and 10.9 kJ for the PL tablet. The tablets could not be distinguished by appearance, taste, or odor and were provided to the participants after concealment.

### 2.5. Whole-Room Indirect Calorie Meter

EE and substrate utilization for each participant were measured in the respiratory chamber. Whole-room indirect calorimeter measurements were obtained by the previously described methods [7]. In brief, the room temperature, humidity, and fresh airflow were set to 25 °C, 40%, and 70 L/minute, respectively. Oxygen consumption (VO_2_) and carbon dioxide production (VCO_2_) were calculated using the method reported by Henning et al. [8]. VO_2_ and VCO_2_ were calculated across a 60-minute period to obtain the values of EE, RQ, fat oxidation, and carbohydrate oxidation for the transient response analysis [9,10]. Protein oxidation was estimated based on urinary nitrogen excretion. All urine samples were collected and weighed while participants were in the whole-room indirect calorie meter and measured in triplicate using a chemiluminescent nitrogen analyzer (TN-100, Mitsubishi Chemical, Kanagawa, Japan).

### 2.6. Blood Samples

Collected blood samples for the measurements as shown in Table 1 were centrifuged at 1000× *g* for 15 minutes at 4 °C to isolate the serum or plasma and were measured by SRL, Inc. (Tokyo, Japan) or LSI Medience Co. (Tokyo, Japan).

### 2.7. Statistics

The primary outcome of this study was the difference in fat oxidation during the night between PL and WA treatments. To determine the effect of WA on the primary outcome, the mean value during the night from 22:00 hour to 06:30 hour was estimated and assessed using a mixed model adjusted by order of the treatment (No significant effect of order of the treatment was observed). A two-sided *p*-value ≤ 0.05 was considered to be statistically significant. All statistical analyses were performed using the IBM SPSS Statistics version 19 (IBM Co., Armonk, NY, USA).

## 3. Results

### 3.1. Characteristics of the Participants

Thirty-five individuals were screened, and 21 were recruited. Of those recruited, one participant dropped out of the study right after the consumption of the PL tablet on the first visit due to an unfavorable feeling due to the texture and flavor of the PL tablet. Twenty participants completed the study and were included in the analyses. From screening until the second visit, no considerable habitual changes were recorded. The characteristics of the participants are presented in Table 1. The dietary records from the three days before the measurements are indicated in Table 2. There were no significant differences in dietary status before the visits or percentage of sleep during the trial between treatments (PL and WA, 92.9 ± 4.3% and 91.9 ± 3.5%, respectively, mean ± standard deviation).

### 3.2. Substrate Utilization

**Figure 2:**
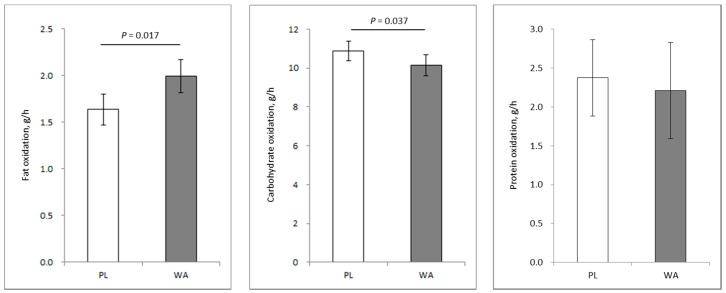
(**Left**) Fat oxidation. (**Middle**) Carbohydrate oxidation. (**Right**) Protein oxidation. Data are means ± standard errors during the night from 22:00 hour to 06:30 hour. Significant differences between the treatments were assessed with a mixed model adjusted by order of treatment.

In the study, the test tablets were ingested 3 minutes before the designated meals, and the substrate oxidation was compared between treatments. The mean fat oxidation during the night increased significantly following WA ingestion as compared with the PL, whereas the mean carbohydrate oxidation significantly decreased. There was no significant difference in the mean protein oxidation (Figure 2). Fat mass is reported to be associated with fat oxidation [11]. In this study, the fat mass before the WA treatment was not significantly different compared with that before the PL treatments (PL and WA, 12.5 ± 2.9 kg and 12.5 ± 3.2, respectively, mean ± standard deviation). The fat oxidation on the fat mass during the WA treatment was significantly increased compared with that during the PL treatment.

### 3.3. EE and RQ

There were no significant differences in the baseline EE (PL and WA, 1189 ± 182 kJ and 1150 ± 156, respectively, mean ± standard deviation) and the baseline RQ (PL and WA, 0.816 ± 0.020 L/L/h and 0.805 ± 0.026, respectively, mean ± standard deviation). There was no significant difference in the mean EE during the night between treatments; however, the mean RQ was significantly lower after WA ingestion than after PL ingestion (Table 3), suggesting a trend in fuel utilization of greater fat oxidation during the night following the WA treatment. Fat free mass change is reported to be associated with EE [12]. In this study, the fat free mass before the WA treatment was not significantly different compared with that before the PL treatment (PL and WA, 49.8 ± 8.2 kg and 49.8 ± 8.0, respectively, mean ± standard deviation). The EE on the fat free mass was also not significantly different between the treatments.

## 4. Discussion

In this study, the effect of the WA treatment on substrate oxidation during the night was investigated in healthy humans. Our previous unpublished study showed a moderate suppressive effect of WA on the glucose response during the night. The underlying mechanism of the effect is thought to be its inhibitory action on alpha-amylase activity [4], which induces a lower carbohydrate absorption and may also accompany the lower glucose-dependent insulinotropic polypeptide (GIP) response. Indeed, the plasma concentration of GIP was lowered significantly by the WA treatment when compared to the PL treatment in our previous unpublished study. A higher postprandial concentration of GIP, an incretin that stimulates insulin secretion from the pancreatic beta-cells, has been directly associated with a lower metabolic rate [13] and stimulates fat accumulation in adipose tissue as an exopancreatic function [14]. Additionally, higher blood GIP levels induced by chronic GIP treatment were shown to reduce fat utilization in high-fat diet-fed mice [15]. Thus, the potential mechanism underlying enhanced fat oxidation by WA treatment may be associated with the GIP lowering effect.

In the present study, fat oxidation was enhanced and RQ was lowered following WA treatment, but EE was not changed. An imbalanced energy balance between energy consumption and the expenditure is directly associated with obesity [16]. Therefore, the anti-obesity effect of tWA via acute increased EE was not expected. However, a low ratio of fat to carbohydrate oxidation was reported to be a predictor of future weight gain in several studies, such as in Pima Indians [1] and healthy women [2]. An assumable underlying mechanism was reported to be enhanced lipogenesis induced by the overflow of carbohydrate metabolism [5]. Therefore, a lowering effect on the ratio of fat to carbohydrate oxidation may reduce the overflow. Although we observed an acute effect on RQ, repeated nightly treatment of WA might have an impact on body fat accumulation overnight. Thus, an intervention study with a focus on weight gain in such patients with night eating syndrome [17] is of interest.

The difference of mean fat oxidation between the treatments was 0.35 g/h (PL and WA, 1.64 ± 0.73 g/h and 1.99 ± 0.79, respectively, mean ± standard deviation). Based on this, the estimated cumulative fat oxidation during the night from 22:00 hour to 06:30 hour (8.5 hours) was 3.0 g. The impact of this amount is estimated to be 1.08 kg as fat under simple calculation if the repeated WA treatment was performed for one year. However, given that there was no difference in energy expenditure, it remains unknown if this would result in any change in body weight or composition.

The limitations and potential biases in this study were the imbalanced gender of the participants (male:female = 14:6) and the use of a single race (Japanese). The included authors are employees of the manufacturer of the test diet.

## 5. Conclusions

WA has the potential to shift the energy balance to a higher ratio of fat to carbohydrate oxidation during the night.

## Figures and Tables

**Table 1:** Characteristics of the participants.

Parameter	Value
Number of participants (male/female)	20 (14/6)
Age, years	38 ± 10
Body weight, kg	62.4 ± 9.8 (M, 67.7 ± 7.7; F, 51.9 ± 4.0)
Body mass index, kg/m^2^	21.8 ± 1.9
Fat mass, kg	12.5 ± 2.9
Fat free mass, kg	49.8 ± 8.2
Systolic blood pressure, mmHg	124 ± 18
Diastolic blood pressure, mmHg	73 ± 14
Glucose, mmol/L	5.36 ± 0.31
Insulin, pmol/L	19.2 ± 7.2
Triglyceride, mg/dL	0.894 ± 0.485

Data are means ± standard deviations. M, male; F, female.

**Table 2:** Dietary records of the three days before the treatment.

Parameter	PL	WA
Energy, kJ/day	8299 ± 1616	8464 ± 1610
Protein, g/day	72.1 ± 18.0	69.5 ± 17.6
Fat, g/day	64.2 ± 19.4	68.7 ± 22.7
Carbohydrate, g/day	249.2 ± 54.5	250.5 ± 46.1

Data are means ± standard deviations. There were no significant differences between treatments. PL: placebo, WA: wheat albumin.

**Table 3:** Energy expenditure (EE) and respiratory quotient (RQ).

Parameter	PL	WA
EE, kJ/h	1148 ± 139	1142 ± 122
RQ, L/L/h	0.904 ± 0.027	0.890 ± 0.032 **

Data are means ± standard deviations during night from 22:00 hour to 06:30 hour. The asterisk denotes significant differences between the treatments assessed with a mixed model adjusted by order of the treatment; ** *p* < 0.01.
